# Impacts of Supplemental Feeding on Sunbird-Pollination Systems in Young Fynbos Varies with Floral Abundance

**DOI:** 10.1007/s00267-024-02089-8

**Published:** 2024-11-16

**Authors:** Asekho Mantintsilili, Sjirk Geerts, Colleen L. Seymour, Anina Coetzee

**Affiliations:** 1 Department of Conservation Management, Nelson Mandela University, George, South Africa; 2 South African National Biodiversity Institute, Kirstenbosch Research Centre, Claremont, South Africa; 3 Department of Conservation and Marine Sciences, Cape Peninsula University of Technology, Cape Town, South Africa; 4 FitzPatrick Institute of African Ornithology, Department of Biological Sciences, University of Cape Town, Rondebosch, South Africa

**Keywords:** Nectarivorous birds, Plant-bird mutualisms, Supplementary feeding, Visitation rate, Young vegetation

## Abstract

Supplementary sugar-water feeding offers nectarivorous birds a source of spatially concentrated food, as opposed to the dispersed food available in flowers. This could impact bird visits to native flowers and alter plant-bird mutualisms, particularly in young post-fire vegetation. This study examined the effects of sugar-water feeders on nectarivorous birds and their plant mutualists in young (burned 18 months previously) and transitional vegetation (burned 5 years previously). A supplementary feeding experiment was conducted at Grootbos Private Nature Reserve in South Africa, using sugar-water feeders when floral abundance was low (winter) and high (spring). We compared bird abundance and visitation rates to flowers before, during, and after feeder presence in both seasons. The use of sugar-water feeders by nectarivorous birds was inversely related to floral abundance, with 679 bird visits (6.94 ± 1.40 bird visitation rate per hour) to feeders in winter and only 90 visits (0.41 ± 0.16 visitation rate per hour) during spring. Bird visits were higher at flowers than at sugar-water feeders, in both seasons. Sugar-water feeders did not influence the visitation rate of sunbirds to flowers in both seasons, contrasting with findings from areas abutting suburbia, suggesting that feeder influence on bird visitation rate may not be apparent in areas with no history of sugar-water feeders. We find that low numbers of feeders do not necessarily compete with natural nectar resources but may instead provide birds with an additional food source, particularly when floral resources are low.

## Introduction

The use of supplementary sugar-water feeders to feed nectarivorous birds is a hobby that has gained popularity worldwide, including in South Africa (Brockmeyer and Schaefer 2012; Sonne et al. 2016; Coetzee et al. 2021; du Plessis et al. 2021). However, concerns are that these sugar-water feeders could disrupt plant-avian pollinator mutualism (Arizmendi et al. 2007). The additional food that supplemental feeding provides to nectar-feeding birds could reduce their visitation to bird-pollinated plants which in turn could have significant negative effects on the reproduction success of their dependent plants (Arizmendi et al. 2007; Sonne et al. 2016). This could interfere with plant-avian mutualisms, particularly in areas with young vegetation where there are few floral resources and bird pollinators (Geerts et al. 2020). Studies indicate that sugar-water feeders support nectar-feeding birds (Pierret and Jiguet 2018; du Plessis et al. 2021), but may also negatively impact their foraging behaviour (Garrison and Gass 1999; Erastova et al. 2023). Van Wilgen (2013) warned that higher human population densities in coastal areas are leading to more frequent fires, reducing the average age of the vegetation, and in recently burned areas, sugar-water feeders are often the only food source available to nectarivorous birds. The interplay between increased burned areas and the use of sugar-water feeders near cities could have a broad range of consequences for the rate of plant recovery, abundance of floral resources available to pollinators, and bird assemblages.

Interacting with nature is thought to promote human well-being (Soga et al. 2016). Due to their often beautifully coloured plumage, nectar-feeding birds such as hummingbirds (Trochilidae) in North and South America, honeyeaters (Meliphagidae) in Australasia, and sunbirds (Nectariniidae) and sugarbirds (Promeropidae) in Africa (Cronk and Ojeda 2008), are desirable in gardens and parks, so feeders are sometimes used to attract them (Arizmendi et al. 2007; Brockmeyer and Schaefer 2012; Sonne et al. 2016; Coetzee et al. 2021; du Plessis et al. 2021). Furthermore, some private nature reserves in the Neotropics use supplementary feeding as a strategy to promote ecotourism, as it provides opportunities for prolonged observations of birds (Brockmeyer and Schaefer 2012). Nevertheless, little is known about how these artificial feeders affect the dynamics of plant-bird mutualisms.

Research has shown that supplementary feeding can help in supporting dry-season bird populations, influencing the abundance and composition of bird communities, and sometimes contributing to their reproductive success (Chamberlain et al. 2007; Pierret and Jiguet 2018; du Plessis et al. 2021). However, the benefits and costs of supplementary feeding for biodiversity overall have been the subject of ongoing debate. This debate arises from conflicting research findings on the impacts of supplementary feeding, with some studies showing a negative, others neutral, or positive effect on bird visits to flowers. Hummingbird studies by Arizmendi et al. (2007) and Ramírez-Burbano et al. (2022) found that hummingbird visits to flowers are reduced when feeders are present, suggesting that feeders lure birds away from flowers. Similar results were reported by du Plessis et al. (2021) on sunbirds and sugarbirds in suburban gardens in South Africa, although an effect was apparent for only one of the two *Erica* species studied. The presence of feeders reduced the visitation rate of sunbirds and sugarbirds to neighbouring flowers of this species, even when floral abundance was high. However, other studies have found that feeders had no overall influence on bird visitation rates to flowers (Inouye et al. 1991; McCaffrey and Wethington 2008), implying they did not deter birds from visiting floral resources. Interestingly, some studies (e.g., Brockmeyer and Schaefer (2012), Sonne et al. (2016)) found increased hummingbird visitation rates to flowers close to feeders, suggesting that feeders may not necessarily compete with flowers but could have a magnet effect (Laverty 1992) to attract birds to flowers in their vicinity.

The current uncertainties regarding the effects of feeders on flower visits are concerning, given the global increase in the use of feeders in gardens, parks, and private nature reserves in recent years (Brockmeyer and Schaefer 2012; Cox and Gaston 2016). While bird visitation may not always guarantee successful pollination (Watts et al. 2012), increased visitation rates generally enhance the likelihood of pollen transfer, and therefore, can sometimes be used as a proxy for pollination (Engel and Irwin 2003). It is also noteworthy that, except for a study carried out in five Ecuadorian forest reserves (Brockmeyer and Schaefer 2012), most supplemental feeding studies carried out worldwide, including in South Africa (Arizmendi et al. 2007; Sonne et al. 2016; Coetzee et al. 2021; du Plessis et al. 2021), have been limited to suburban areas. The complexity of suburban habitats may impact how frequently birds visit feeders and flowers. Furthermore, supplemental feeding has been a common practice in suburban areas; thus, urban-adapted birds are more likely to be accustomed to feeders and, hence, readily respond to them. Therefore, this study proposes that to fully comprehend the effects of supplementary feeding on plant-avian pollinator mutualisms, this phenomenon should be evaluated in a natural setting where supplementary feeding has never occurred, and birds rely exclusively on natural vegetation for resources. Moreover, since supplemental feeding has recently been introduced in private nature reserves elsewhere and has the potential to gain popularity (Brockmeyer and Schaefer 2012), particularly with growing ecotourism, this study could help inform policy about supplemental feeding, particularly for conservation agencies in the Cape Floristic Region (CFR).

The CFR is one of the smallest, yet most biologically diverse regions in the world and is known for its high levels of plant diversity and endemism (Goldblatt and Manning 2002). It is home to over 9000 flowering plant species, with 69% of these endemic to this region (Goldblatt and Manning 2002). These include species from well-known families such as the Proteaceae, Ericaceae, and Orchidaceae, the majority of which exclusively rely on animals (e.g., birds, insects, and others) for pollen transfer. Consequently, plant-pollinator mutualisms in the CFR play a key role in the reproductive success of many plant species (Van der Niet and Johnson 2009). Birds pollinate at least 300 plant species (3%) of the CFR flora and are thus crucial to the plant diversity of this region (Rebelo 1987; Geerts et al. 2020). Surprisingly, only four nectar-specialist bird species (birds feeding predominantly on nectar) occur throughout this region: the Cape sugarbird *Promerops cafer*, Orange-breasted sunbird *Anthobaphes violacea*, Malachite sunbird *Nectarinia famosa*, and Southern double-collared sunbird *Cinnyris chalybeus* (Pauw and Johnson 2018; Geerts et al. 2020). Since the CFR is fire-prone and well-adapted to fire, it is recommended that this region burn every 12 to 15 years for conservation purposes, with longer fire return intervals in dry areas (Kraaij and Van Wilgen 2014; Geerts 2021). In comparison to mature vegetation, young fynbos (i.e., burnt patches) often has fewer floral resources and bird pollinators (Chalmandrier et al. 2013; Geerts et al. 2020). Consequently, the presence of sugar-water feeders in a post-fire environment may increase the susceptibility of bird-pollinated plants to reduced pollinator visits since feeders offer nectar-feeding birds a plentiful food supply.

This study explores the potential implications of supplemental feeding on plant-pollination mutualism in young vegetation. In a non-urban environment with no history of supplementary feeding, we ask: (i) Does supplementary feeding draw nectarivorous birds away from the surrounding vegetation? (ii) If so, which vegetation—young or transitional—would be more vulnerable? And lastly, (iii) does the introduction of sugar-water feeders affect the visitation rate of nectarivorous birds to surrounding flowering plant species? Given that supplemental feeding has never been done in the natural landscapes of the CFR, we predicted that sugar-water feeders would either increase flower visits or be neutral (no effects). We also predicted that the vegetation that had burned eighteen months previously (henceforth the “young vegetation”) would show greater effects of feeders than the vegetation burned five years previously (henceforth the “transitional vegetation”), given that more recently burned vegetation typically has fewer floral resources (Geerts et al. 2020).

## Methods

### Study Area

**Fig. 1:**
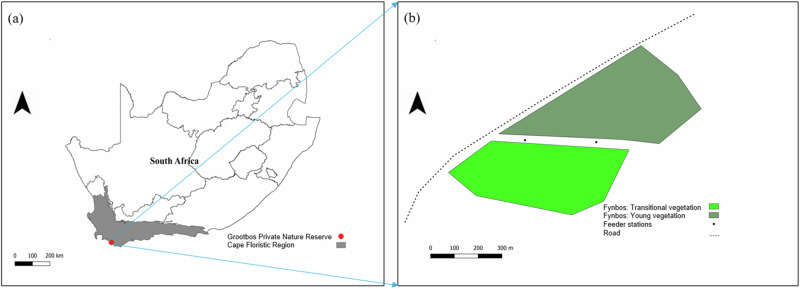
The location of Grootbos Nature Reserve (red dot) in the Cape Floristic Region (CFR) of South Africa is shown in **a**. The extent of the young and transitional vegetation in the study site is shown in **b**, along with the locations of the two feeder stations (black dots). The two vegetation age categories were surrounded by transitional fynbos vegetation (i.e., burned more than 5 years ago), covering over a hectare around the two vegetation types

To assess the effects of supplemental feeding on nectarivorous birds and the plants they pollinate in a post-fire environment, an experiment with supplementary feeding was carried out using a matched-pairs design in young (burned 18 months previously) and transitional vegetation (burned more than 5 years previously) in the Grootbos Private Nature Reserve (GPNR) in South Africa. The GPNR (34°32'30” S; 19°24'50” E) is located a few kilometres from Gansbaai in the Western Cape of South Africa, along the western edge of the Agulhas Plain in the Cape Floristic Region (Fig. 1a). It is a popular tourism site in the Overberg District. This reserve extends over 2500 hectares (25 km²) of pristine wilderness and is home to more than 800 plant species, 100 of which are threatened; three of the threatened plant species are endemic to this reserve (Dube and Nhamo 2021). Fynbos shrubland (95.3%) dominates the GPNR’s vegetation, with a few patches of forest (4.6%) and wetland (0.1%). The climate in this region can be described as Mediterranean, with significant seasonal variations in precipitation. On average, June is the wettest month (~88 mm of precipitation), while January is the driest, with ~27 mm of precipitation, and average annual rainfall is 614 mm. The daily maximum temperatures range between 27 °C in February and 19 °C in July. The GPNR is frost-free. Supplementary sugar-water feeding has never been practised in the GPNR.

The supplementary feeding experiment was carried out during winter (July/August) 2023 in young (22.9 ha) and adjacent transitional (9.2 ha) fynbos vegetation within the GPNR (Fig. 1b). In both vegetation age categories, the floral abundance of flowering plant species pollinated by nectarivorous birds was relatively low in winter compared to spring. The experiment was repeated in spring (September/October 2023), when floral abundance was much higher. Surveys were conducted over the two seasons to determine if feeder influence varies with seasonality and floral abundance. The two vegetation age categories were surrounded by transitional fynbos vegetation, which had also been burned more than five years previously. However, both vegetation types were separated from the surrounding vegetation by a 30-m wide firebreak on all sides, and also a road on one side. As a result, we chose to sample exclusively within these two large areas. Restionaceae such as *Elegia thyrsifera* dominated both vegetation age categories. Bird-pollinated flowers present were *Satyrium carneum*, *Salvia africana-lutea*, and *Lessertia frutescens*. Nectar specialist bird species occurring in these two vegetation age categories include the Amethyst sunbird *Chalcomitra amethystina*, Malachite sunbird *Nectarinia famosa*, and Southern double-collared sunbird *Cinnyris chalybeus* (AM unpublished data). Nectar generalist species such as the Cape white-eye *Zosterops virens*, Cape weaver *Ploceus capensis*, and Cape bulbul *Pycnonotus capensis* also occur.

### Flowering Species

This study aimed to assess the impact of supplementary feeding on floral visitation to three bird-pollinated plant species during peak flowering: *S. africana-lutea* (winter: July/August 2023), *L. frutescens* (spring: September/October), and *S. carneum* (spring: September/October 2023). *Lessertia frutescens* was only flowering in the transitional vegetation, whereas *S. africana-lutea* and *S. carneum* were flowering in both young and transitional vegetation. Inflorescence and floral abundance of these flowering plant species were counted in both young and transitional vegetation during both seasons to estimate the nectar resources available for nectar-feeding birds in the surrounding vegetation. To estimate the inflorescence and floral abundance of *S. carneum*, five 10 × 10 m plots were placed randomly in four patches containing bird-pollinated flowers next to each of the two chosen points in young and transitional vegetation (i.e., 50 and 200 m from the sugar-water feeders). All *S. carneum* inflorescences and flowers found within each plot were counted. Since the two winter flowering species (*L. frutescens* and *S. africana-lutea*) were not densely flowering, we counted all flowers within a 30 m radius of each chosen point per vegetation.

We also measured the nectar standing crop (both volume and concentration) of three open flowers in 15 inflorescences of both *S. carneum* and *L. frutescens*. The nectar volume was measured with a 10 mL capillary tube, and nectar concentration with a handheld refractometer (Bellingham and Stanley, Tunbridge Wells, UK). The nectar values of *S. africana-lutea* were obtained from Mnisi et al. (2021). All the measured nectar values were converted to milligrams of sucrose per flower, and the total amount of sucrose produced by inflorescences per hectare was determined from these data.

### Bird Abundance, Richness, and Visitation Rate

#### Pre-experimental phase

As a control for the experiment, 24 sessions (i.e., 12 sessions for each vegetation age category) of direct observations (20 min per session) were conducted in the two vegetation age categories using a pair of Bushnell binoculars to observe nectarivorous bird-plant interactions for three days. Two points—one close to the sugar-water feeders (50 m) and the other further away (200 m)— in each of the two vegetation age categories, and four patches of bird-pollinated plant species closest to each point were identified as focal patches. All the chosen focal patches were at least 20 m away from each point. For each point, all nectarivorous bird visitors (i.e., nectar specialists and generalists) seen in focal patches were identified to species level and their abundance (i.e., maximum number seen at a time), sex, age (juvenile or adult) and the number of visits to the flowers were also recorded. All observations were conducted in the morning (before 9 a.m.) and in the afternoon (after 3 p.m.), when foraging activity was at its peak. Rainy, cloudy, and very windy days were avoided.

#### Experimental phase

On day four, eight nectar feeders filled with a 20% (weight/weight) sucrose solution, commonly used in supplementary feeding (du Plessis et al. 2021), were placed on the 30 m broad firebreak at two stations (each with 4 feeders x 2 = 8 feeders). The two feeder stations were >200 m apart. All sugar-water feeders were cleaned with detergent and refilled with the sugar-water solution daily. A week later, six 20-minute observations were carried out again at each of the same points that were monitored before the experiment in nearby young and transitional vegetation, including at feeder stations for three days. The abundance of nectarivorous birds observed in focal patches, their sex, and number of visits to the flowers were also noted during the experimental phase for each point, as with the pre-experimental phase. Furthermore, the same variables were also recorded for birds at feeders during this phase.

#### Post-experimental phase

All sugar-water feeders were emptied on the last day of the experiment and left for a day in the same position at the two feeder stations. Two days later, the same variables (e.g., nectarivorous bird visitors and their abundance) recorded in the pre-experimental and experimental phases were recorded for the next three days. The same locations in nearby young and transitional vegetation that had previously been observed were also observed during this phase, including empty artificial feeders. Spring sampling was conducted by two independent observers simultaneously, resulting in 64 h of bird observations. The winter sampling was conducted by a single observer, which resulted in 32 h of bird observations. It is acknowledged that since sunbirds frequent the same area, it is likely that the recorded birds were the same individuals.

### Data Analysis

#### Bird abundance

All data exploration and analyses were performed in R (version 4.3.0) (R Core Team 2023). We used Generalised Linear Mixed Models (GLMMs) for the winter and spring datasets separately to test whether feeders drew nectarivorous birds away from the surrounding vegetation. For bird abundance, GLMMs with a Poisson error distribution were constructed with the *lme4* package (Doran et al. 2007). We ran models using all combinations of explanatory variables “floral abundance” (scaled), “treatment” (feeders, young, and transitional vegetation) and “timeframe” (pre-, experimental, and post-experimental), with feeder station (station 1 and 2) as a random intercept and sensible interactions between them (Table S1). An information theoretic approach was then used to choose the best model. We tested for zero-inflation and overdispersion using the *DHARMa* package (Hartig 2018) and ranked all candidate models using the corrected Akaike Information Criterion (AICc) and AICc weights (*w*_*i*_) through the *AICcmodavg* package (Burnham et al. 2011; Mazerolle 2019). The model with a lower value of AICc (<2 compared to others) was identified as the best model.

#### Visitation rate

To determine if the introduction of feeders impacted bird visitation rate, we constructed Generalised Linear Mixed Models (GLMMs) for *S. africana-lutea* and for *L. frutescens* and *S. carneum* combined, because there were few records of bird visits to *L. frutescens* (i.e., only five records across the spring survey). We used a Gaussian distribution (link = “identity”) in the *glmmTMB* package (Brooks et al. 2022). The visitation rate was calculated as bird visits per flower per hour. A total of four candidate models were run, with the base model including the response variable, bird visitation rate and predictor variables “floral abundance” (scaled), “treatment” (feeders, young, and transitional vegetation) and “timeframe” (pre-, experimental, and post-experimental), with feeder stations (station 1 and 2) as random intercept (Table S1). The best model fit was chosen using the AICc through the *AICcmodavg* package (Mazerolle 2019), in the same way as described for bird abundance. Finally, we performed pairwise comparisons to examine specific interactions between fixed variables using the emmeans function in the *emmeans* package for both bird abundance and visitation rate. The floral abundance between young and transitional vegetation was statistically compared using a two-sided, independent samples t-test for both seasons.

The normalised residuals were plotted against the fitted values in scatter plots to assess model fit. Variance Inflation Factors were used to assess collinearity among fixed variables. We determined the variance explained by the fixed and random effects by deriving marginal (R^2^m) and conditional pseudo-R^2^ values (R^2^c; Nakagawa and Schielzeth 2013) using the “r.squared GLMM function” from the *MuMIn* package (Barton 2015).

## Results

### Flowering Species

In winter, when floral abundance was relatively low, inflorescence abundance was significantly higher in transitional (mean ± SE: 0.23 ± 1.19 per m^2^, *n* = 133) than in young vegetation (0.16 ± 0.05 per m^2^, *n* = 54) (*t* = 2.21, *p* < 0.0005). In contrast, inflorescence abundance was significantly higher in young (2.24 ± 0.07 per m^2^, *n* = 467) than in transitional vegetation (1.23 ± 0.19 per m^2^, *n* = 341) in spring, when floral abundance was high (*t* = 0.94, *p* = 0.003). Nectar density was higher in spring (mean ± SE: 15 ± 0.04 g of sucrose per ha^−1^) than winter (6 ± 0.02 g of sucrose per ha^−1^).

### Bird Abundance and Richness

In winter, we recorded 1376 bird visits in the surrounding vegetation within the two vegetation age categories, by four specialists and three opportunistic nectar feeders. Of the observed specialists, only two species were common between the two vegetation age categories, *Cinnyris chalybeus* and *Nectarinia famosa*. These two species were also the only ones observed at the feeders, with *C. chalybeus* dominating both vegetation age categories and at feeders (Table 1). Of the visiting birds, when floral abundance was low, 81.4% were adults and 18.6% were juveniles. When floral abundance was high (i.e., spring), we recorded 3384 bird visits by three specialists and three opportunistic nectar-feeding species. Similarly, *C. chalybeus* and *N. famosa* were the only bird species recorded at feeders in times of high floral abundance, with *C. chalybeus* being the most frequently observed species in both vegetation age categories and at feeders (Table 1). Most (99.2%) of the visiting birds were adults, while only 0.8% were juveniles when floral abundance was high. Interestingly, none of the recorded opportunistic nectar feeders were observed at the feeders throughout the study.

**Table 1:** The abundance of nectar specialists (in bold) and opportunistic nectar feeders encountered during winter (low floral abundance) and spring (high floral abundance) sampling at feeder stations, young, and transitional fynbos vegetation at GPNR (**-**species not observed)

Species	Young vegetation	Transitional vegetation	Feeders	Total
**Winter**				
***Cinnyris chalybeus***	209	333	45	587
***Nectarinia famosa***	18	32	27	77
*Zosterops virens*	19	17	–	36
*Pycnonotus capensis*	31	39	–	70
*Ploceus capensis*	19	30	–	49
***Anthobaphes violacea***	–	1	–	1
***Promerops cafer***	–	1	–	1
**Total**	**296**	**453**	**72**	**821**
**Spring**				
***C. chalybeus***	409	323	3	735
***N. famosa***	63	34	3	100
*Z. virens*	44	31	–	75
*P. capensis*	40	73	–	113
*Ploceus capensis*	21	28	–	49
***Chalcomitra amethystina***	13	7	–	20
**Total**	**591**	**496**	**6**	**1092**

**Fig. 2:**
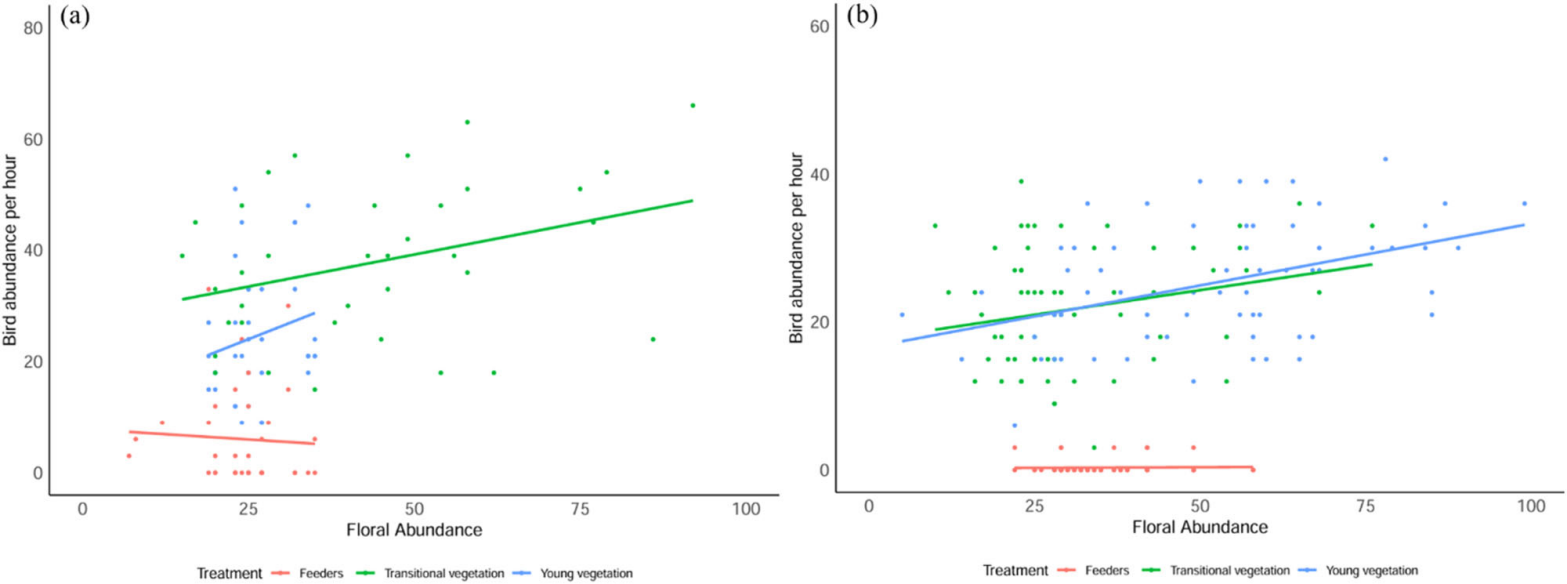
The abundance of nectarivorous birds recorded visiting sugar-water feeders and flowers in young and transitional vegetation per hour during the feeding experiment at GPNR in winter (**a**) and spring (**b**). Note: (**a**) = 32 h and (**b**) = 64 h of observations. Dots represent the abundance of nectar-feeding bird visits to flowers and sugar-water feeders

Overall bird abundance was strongly influenced by the interaction of treatments (feeders, young, and transitional vegetation), floral abundance and timeframe (pre-, experimental, and post-experimental phase) in both times of low and high floral abundance (Table S1 for detailed stats: *w*_*i*_ = 100%, R^2^m = 0.64, R^2^c = 0.68 and *w*_*i*_ = 100%, R^2^m = 0.58, R^2^c = 0.66, respectively). Distance from the sugar-water feeders did not significantly influence the overall abundance and visitation rate of nectarivorous birds to flowers in both vegetation age categories and both seasons. As a result, it was excluded from further analysis. Feeders increased overall bird abundance in transitional vegetation, but only during winter, with no effects on young vegetation for both seasons. The number of nectarivorous birds at feeders decreased with increased floral abundance, while bird numbers to flowers increased with increased floral abundance in both seasons (Fig. 2a, b).

Despite receiving 679 bird visits, sugar-water feeders had significantly fewer bird visits than flowers during the experimental phase in both young and transitional vegetation in winter (Table 2). The number of nectarivorous birds did not differ significantly between the pre- and experimental phases in the young vegetation (Table 2). However, overall bird abundance significantly increased in the transitional vegetation when sugar-water feeders were present, and that effect was stronger during winter (Table 2). In winter, a few birds (16 visits by *N. famosa* and 18 by *C. chalybeus*) continued to visit feeders after the experiment had ended (during the post-experimental phase) (Fig. S1).

**Table 2:** Results from the best fitting model for each season after model selection to determine which variables best predict the abundance of nectarivorous bird species at the sugar-water feeders, in the young and transitional fynbos vegetation at GPNR

Bird abundance	Estimate	SE	t-ratio	*P*
*Winter*				
Intercept	−0.31	0.14	−2.25	0.002*
Feeders B: young vegetation B	−2.41	0.20	−12.23	< 0.0001*
Young vegetation A: young vegetation B	0.04	0.06	2.35	0.23
Feeders B: transitional vegetation B	−2.05	0.19	−10.65	< 0.0001*
Transitional vegetation A: transitional vegetation B	−0.36	0.05	−4.21	0.01*
*Spring*				
Intercept	0.15	0.33	0.46	< 0.0001*
Feeders B: young vegetation B	−4.24	0.25	−16.99	< 0.0001*
Young vegetation A: young vegetation B	0.01	0.04	0.12	0.32
Feeders B: transitional vegetation B	−4.17	0.25	−16.71	< 0.0001*
Transitional vegetation A: transitional vegetation B	0.05	0.04	1.21	0.95

The parameter estimates, standard errors (SE), *t*-values, and *P*-values for each variable are presented. A colon (:) indicates interactions between variables, A = pre-experimental phase, B = experimental phase, and significant *P*-values are in bold and indicated with an asterisk (*). Comparisons are made between the pre-experimental and experimental phases only; data from the post-experimental phase are not included.

Similarly, in spring, sugar-water feeders had significantly fewer nectarivorous bird visits than flowers during the experimental phase in both vegetation age categories (Table 2). Sugar-water feeders received only 90 bird visits in spring. In contrast, there was no significant difference in overall bird abundance when comparing the pre-and experimental phases in both the young and transitional vegetation (Table 2), suggesting that sugar-water feeders did not influence overall bird abundance during spring. Only one species, *C. chalybeus*, was observed visiting feeders during the post-experimental phase when floral abundance was high (Fig. S1).

### Visitation Rate

**Fig. 3:**
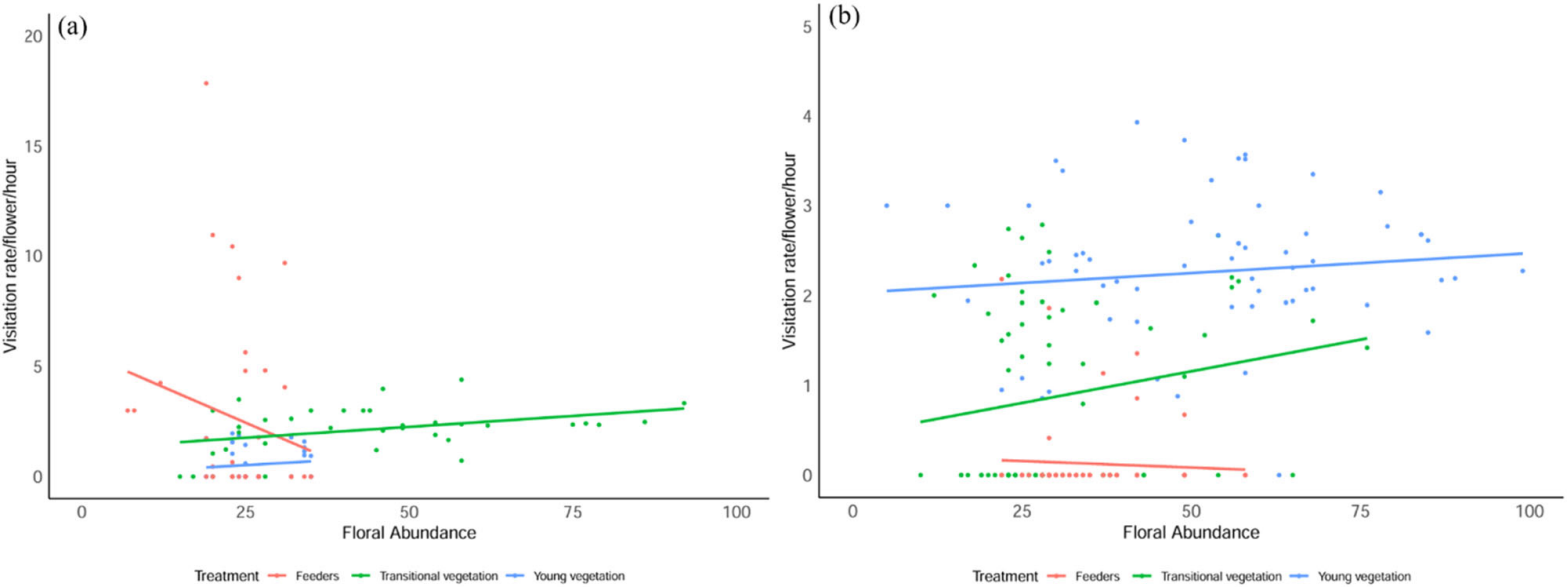
The visitation rate of nectarivorous birds to sugar-water feeders and the surrounding young and transitional vegetation per flower per hour during the feeding experiment at GPNR in winter (**a**) and spring (**b**). Note: (**a**) = 32 h and (**b**) = 64 h of observations. Dots represent bird visits to flowers and feeders

The overall floral visitation rate for both seasons was strongly influenced by the three-way interaction of floral abundance, timeframe (pre-, experimental, and post-experimental) and treatment (feeders, young, and transitional vegetation) (Table S1: *w*_*i*_ = 99%, R^2^m = 0.60, R^2^c = 0.62 and *w*_*i*_ = 100%, R^2^m = 0.47, R^2^c = 0.47, respectively). The introduction of sugar-water feeders did not affect the overall visitation rate of nectarivorous birds to neighbouring flowers in both the young and transitional vegetation throughout the study. Bird visitation rate at feeders decreased with increased floral abundance, while their visitation rate to flowers increased with increased floral abundance in both seasons (Fig. 2a, b). The number of feeder visits by nectar-feeding birds was inversely related to floral abundance (Fig. 3a, b).

In winter, when there was little floral resource, sugar-water feeders had significantly greater bird visitation rate than flowers during the experimental phase in both the young and transitional vegetation (Table 3). Feeders had an average of (mean ± SE) 6.94 ± 1.40 bird visits per hour during this phase, while the young and transitional vegetation received an average of 0.55 ± 0.21 and 2.84 ± 0.64 visits per flower per hour, respectively. However, there was no significant difference in bird visitation rate to flowers between the pre- and experimental phase in both the young and transitional vegetation during winter (Table 3).

**Table 3:** Results from the best fitting model for each season after model selection to determine which variables best predict the visitation rate for nectarivorous bird species to the sugar-water feeders and flowers in the young and transitional fynbos vegetation at GPNR

Visitation rate	Estimate	SE	t-ratio	*P*
*Low floral abundance*				
Intercept	0.41	0.91	3.72	0.002*
Feeders B: young vegetation B	4.55	0.86	5.30	< 0.0001*
Young vegetation A: young vegetation B	0.02	1.01	0.03	0.99
Feeders B: transitional vegetation B	3.41	0.92	3.72	0.01*
Transitional vegetation A: transitional vegetation B	0.45	1.16	0.39	0.43
*High floral abundance*				
Intercept	0.62	0.37	1.66	< 0.0001*
Feeders B: young vegetation B	0.73	0.41	1.81	< 0.0001*
Young vegetation A: young vegetation B	−0.52	0.24	−2.07	0.49
Feeders B: transitional vegetation B	−0.55	0.36	−1.52	**0.04**
Transitional vegetation A: transitional vegetation B	−0.43	0.24	−1.83	0.67

The parameter estimates, standard errors (SE), *t*-values, and *P*-values for each variable are presented. A colon (:) indicates interactions between variables, A = pre-experimental phase, B = experimental phase, and significant *P*-values are in bold and indicated with an asterisk (*). Comparisons are made between the pre-experimental and experimental phases only; data from the post-experimental phase are not included.

In contrast, when floral abundance was high in spring, sugar-water feeders received significantly fewer bird visits than flowers during the experimental phase in both young and transitional vegetation (Table 3). During this phase, sugar-water feeders had an average of 0.41 ± 0.16 bird visits per hour, while young and transitional vegetation received an average of 2.65 ± 0.22 and 0.97 ± 0.21 bird visits per flower per hour, respectively. Bird visitation rate to flowers did not differ significantly between the pre- and experimental phases in the young and transitional vegetation in spring (Table 3). The most frequent visitors to flowers were *C. chalybeus* (accounted for 46% of all visits), followed by *N. famosa* (32%) throughout the study.

## Discussion

We found that supplementary feeding can alter bird abundance but does not necessarily affect their visitation rates to surrounding flowering plants in a natural setting, but these impacts vary with vegetation maturity and floral abundance.

### Bird Abundance

Similar to previous studies on hummingbirds (Avalos et al. 2012; Brockmeyer and Schaefer 2012; Sonne et al. 2016) and on sunbirds (du Plessis et al. 2021), we found that sugar-water feeders affected the relative abundance of nectar-feeding birds, but this effect varied with season (Inouye et al. 1991; Avalos et al. 2012), and vegetation age.

In winter, when floral abundance was low, sugar-water feeders received more bird visits (679 bird visits; 6.94 ± 1.40 bird visits per hour) than in spring (90 visits; 0.41 ± 0.16 visits per hour), when floral abundance was high in the surrounding vegetation. Similar observations have been made for hummingbirds (Inouye et al. 1991; Avalos et al. 2012), which used feeders more frequently during seasons with low floral abundance than in seasons with high floral abundance. In contrast to the large volumes of nectar that birds receive from feeders, *S. africana-lutea*, the only bird-pollinated species flowering in winter, was sparsely flowering and produced little nectar sugar per hectare (6 g of sucrose per ha^−1^). In addition, this species produces dilute, bitter-tasting nectar (Wester and Claßen-Bockhoff 2006; Mnisi et al. 2021). These could account for the high number of nectar-feeding birds observed at sugar-water feeders in winter. Furthermore, since the abundance of nectar-feeding birds is strongly influenced by nectar volume at small spatial scales (Geerts et al. 2020), sugar-water feeders might have provided birds with an essential food source, leading to increased use in times of low floral resources. We could also expect few nectar-feeding birds at feeders relative to the surrounding flowers in typical fynbos (i.e., fynbos with high volumes of *Protea* nectar), particularly in winter. Bird assemblages may not be impacted by artificial feeders in typical fynbos since they may have access to plentiful natural nectar sources.

Nectar-feeding birds used sugar-water feeders less in spring, suggesting that as the seasons change and natural food sources become more abundant, birds may rely less on feeders to supplement their diet (Inouye et al. 1991; McCaffrey and Wethington 2008). The profuse flowering of *S. carneum* in spring might have provided birds with more concentrated nectar compared to feeders or a source of nectar with which they were more familiar, given that birds in this setting were fairly naïve to sugar-water feeders. The flowers of this species produce large volumes of concentrated nectar (23–32.5%) (Johnson 1996). Species such as *N. famosa* prefer nectar sources with high sugar concentrations (Brown et al. 2010) and bird search images for flowers may be more dominant than those for sugar-water feeders. In addition, there was high nectar sugar (15 g of sucrose per ha^−1^), floral abundance and a variety of nectar resources available for nectar-feeding birds in the surrounding vegetation in spring. It is therefore not surprising that birds were attracted in high numbers to these flowering species compared to sugar-water feeders in spring. In addition, sugar-water feeders offer birds a large amount of spatially concentrated food, increasing competition and interspecies conflict (Campbell 2010; Le Louarn et al. 2016), whereas flowers are dispersed and provide a variable resource across territories. These results are supported by findings in studies on hummingbirds (Inouye et al. 1991; McCaffrey and Wethington 2008; Brockmeyer and Schaefer 2012), who reported a negative correlation between flower abundance and feeder visits. Although the present study took place at a single study site (GPNR), the area is large, and the model results suggest that much of the observed variation is well explained by the explanatory variables.

The young vegetation was predicted to show a greater effect of sugar-water feeders than the transitional vegetation, due to less floral resources in this vegetation. Instead, when floral abundance was low (i.e., in winter), feeders increased bird numbers in transitional vegetation, with no significant impacts on young vegetation. Interestingly, this coincided with higher floral abundance at the transitional vegetation compared to the young vegetation during this period. These results suggest that feeders attract more birds to areas with already abundant natural resources. This could be linked to the high floral resources and vegetation structure available in the transitional vegetation compared to the young vegetation. The neutral effects of feeders on bird abundance in young vegetation, when floral abundance was low, could be explained by the relatively low bird numbers in the young vegetation, possibly due to the limited structural resources (i.e., vegetation structure) available for their survival (Geerts et al. 2012). Overall, these results suggest that sugar-water feeders do not necessarily replace the natural food resources available for birds, but rather provide them with an additional food source, particularly when floral resources are scarce.

### Bird Species Richness

Interestingly, despite the presence of opportunistic nectar feeders and other specialist nectarivore birds in the surrounding vegetation, only two specialist nectarivore birds (*C. chalybeus* and *N. famosa*) visited feeders. Similar to a study by du Plessis et al. (2021) on sunbirds, *C. chalybeus* was the most abundant species at the sugar-water feeders. These findings could be explained by the generally high prevalence of these two species in the surrounding vegetation. On the other hand, the large-bodied *N. famosa* requires large nectar resources and is associated with long tubular flowers (e.g., Iridaceae and Ericaceae species) (Geerts and Pauw 2009). The absence of long tubular flowers with large nectar resources in times of low floral resources could explain the high prevalence of this bird species at sugar-water feeders.

In contrast to findings by du Plessis et al. (2021), sugar-water feeders did not attract *Pycnonotus capensis*, *Ploceus capensis*, *Z. virens*, *P. cafer* and *A. violacea* in a natural setting. *Pycnonotus capensis*, *Ploceus capensis*, and *Z. virens* are opportunistic nectar feeders, with diverse diets, including insects, fruits, and nectar. Therefore, their absence at feeders could be because the surrounding vegetation may have provided sufficient food sources for them, reducing the need to visit sugar-water feeders. The two nectar specialist birds (*P. cafer* and *A. violacea*) were rare in the surrounding vegetation (only one individual of each species was observed), occurring only in old fynbos vegetation further away from the focal vegetation. *P. cafer* is strongly linked to bird-pollinated Proteaceae shrubs (Geerts et al. 2020). The absence of these shrubs in the nearby surrounding vegetation could explain their relative absence. Moreover, since the GPNR has no history of bird feeders, most nectarivorous birds would not have been familiar with feeders as a food source. This contrasts with urban areas, where birds are accustomed to feeders and may even rely on them and so respond quickly to their presence.

### Bird Visitation Rate

Despite receiving many bird visits in winter and a few in spring, sugar-water feeders did not significantly influence bird visitation rates to flowers in the young and transitional neighbouring fynbos vegetation. These findings contrast with work on hummingbirds (Arizmendi et al. 2007; Avalos et al. 2012; Sonne et al. 2016) which found increased or reduced bird visitation rates to nearby flowers in the presence of sugar-water feeders. In addition, a study by du Plessis et al. (2021) on sunbirds and sugarbirds in South Africa also reported a reduced visitation rate to flowers in the presence of feeders, although an effect was apparent for only one of the two *Erica* species studied. These contrasting results could be explained by several factors.

Firstly, sugar-water feeders were present for ten days in this study, but this may have been insufficient time for birds to become accustomed to feeders. Other studies, e.g., Sonne et al. (2016) used feeders for extended periods. Others, where feeders were only available for a week (e.g., Du Plessis et al. 2021), or even a single day (Arizmendi et al. 2007), or two days (Avalos et al. 2012), saw almost immediate responses by birds to sugar-water feeders. This is likely because these studies all took place in areas with a history of feeder use. Secondly, the present study was conducted in a natural setting with no history of feeder use; whereas supplemental feeding is common in suburban areas; thus, urban-adapted birds are more likely to be accustomed to feeders and, hence, be easily attracted by them. We used eight feeders while other studies such as Sonne et al. (2016) used a dozen feeders (not stated) and Du Plessis et al. (2021) used four feeders per garden in 18 gardens.

The findings of this study suggest that low numbers of sugar-water feeders may not necessarily disrupt the mutual relationship between birds and pollinators in areas where birds are not accustomed to sugar-water feeders. However, an effect may become apparent if birds become familiar with sugar-water feeders, potentially disrupting plant-bird mutualisms. To fully understand the impacts of sugar-water feeders on plant-bird mutualism, we recommend that future studies use a higher density of feeders. Some studies could assess the time it takes different species to adapt to sugar-water feeders and which species are the “early adopters”, while others could explore how this might affect plant-bird pollination in the surrounding native vegetation. It would also be valuable to explore the relative effect of sugar-water feeders on the distribution of bird visits across individual flowers, particularly in settings of low flower abundance. This could help determine whether, per flower, bird visits to flowers might increase or remain stable in the presence of sugar-water feeders. Understanding these dynamics could help improve our knowledge of the implications of supplementary feeding on plant-bird mutualism.

## Conclusions

In conclusion, we found the use of sugar-water feeders by nectarivorous birds to be inversely related to floral abundance in an area where birds had had no previous exposure to sugar-water feeders, suggesting that naïve birds preferred flowers to feeders. Where birds are accustomed to feeders, there may be a more marked response in favour of feeders. Our findings show that feeders do not necessarily replace or compete with the natural nectar resources available for birds but may rather supplement their food source. However, to fully understand the potential implications of supplemental feeding on the plant-pollination mutualism in young vegetation, future studies should consider multiple locations, with different feeder histories.

## Supplementary information

Fig S1

Table S1

## Data Availability

The data that support the findings of this study are available from the corresponding author upon request.
