# Supplementary material for: Impacts of Supplemental Feeding on Sunbird-Pollination Systems in Young Fynbos Varies with Floral Abundance
Source: Environ Manage. 2024 Nov 16;75(4):906–17. doi: 10.1007/s00267-024-02089-8 (PMC11965216; doi:10.1007/s00267-024-02089-8)
Supplement: Supplementary file 1 — Fig S1 [file 267_2024_2089_MOESM1_ESM.docx]

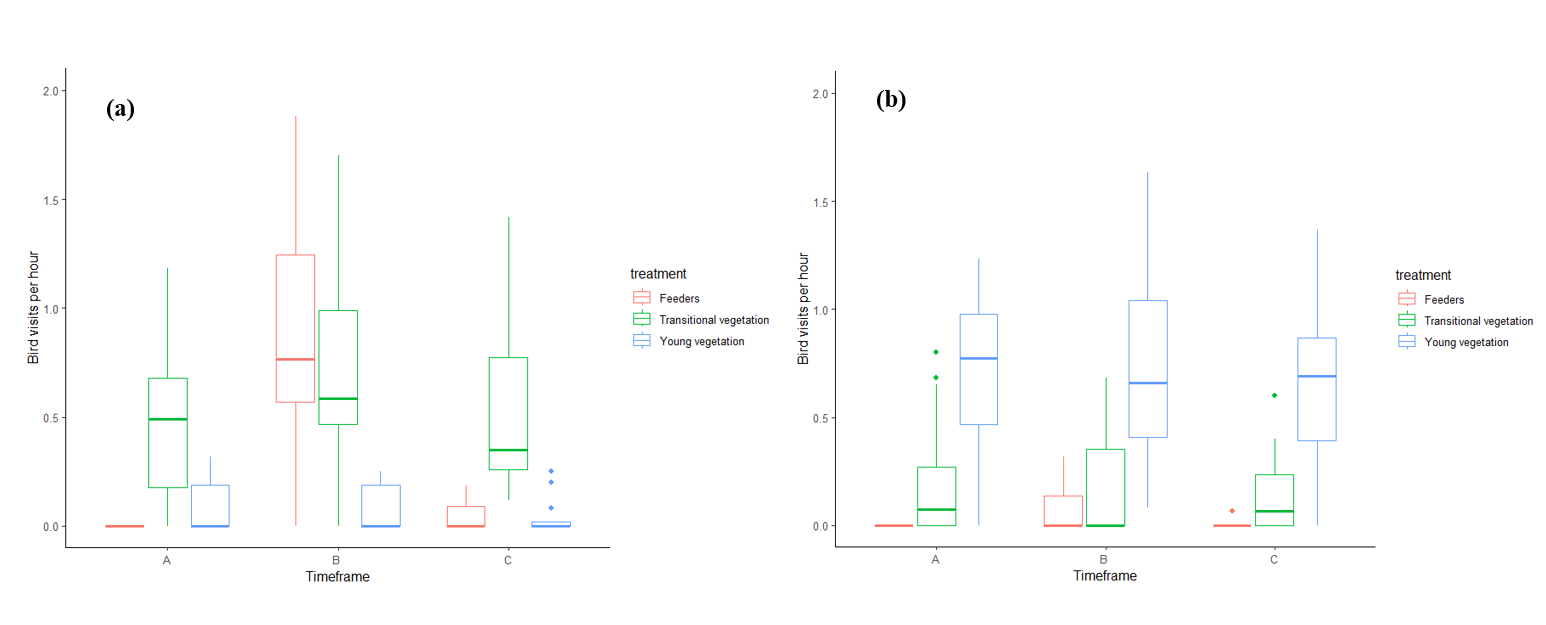


**Fig. S1** Sunbirds visit per hour at the feeders, in young, and transitional vegetation throughout the (a) winter and (b) spring pre- (A), experimental- (B), and post-experimental phases (C) of the feeding experiment at GPNR. Box plots show variations in bird visits, with lines in the middle indicating means, whiskers indicating variability outside the lower and upper quartiles and dots represent outliers.
