# Supplementary material for: Impacts of Supplemental Feeding on Sunbird-Pollination Systems in Young Fynbos Varies with Floral Abundance
Source: Environ Manage. 2024 Nov 16;75(4):906–17. doi: 10.1007/s00267-024-02089-8 (PMC11965216; doi:10.1007/s00267-024-02089-8)
Supplement: Supplementary file 2 — Table S1 [file 267_2024_2089_MOESM2_ESM.docx]

**Table S1** A summary of all models with predictor variables predicting the abundance and floral visitation rate of nectarivorous birds in two vegetation types (young and transitional) during winter (low floral abundance) and spring (high floral abundance) at GPNR. For each model, the number of parameters (K), log likelihood (L), Akaike information criterion (AICc), difference in AICc from the best model and the Akaike weight (*wi*) is presented. An asterisk (*) indicates interactions between variables. Timeframe includes pre-, experimental and post-experimental phases, while treatments include feeders, young and transitional vegetation

| **Season** | **Response variable** | **Model** | **K** | **L** | **AICc** | **ΔAICc** | ***wi*** |
| --- | --- | --- | --- | --- | --- | --- | --- |
| Low floral abundance | Abundance | timeframe * treatment * floral abundance | 10 | -428.21 | 878.6 | 0.00 | 1 |
|  |  | timeframe + treatment * floral abundance | 8 | -540.28 | 1098.00 | 219.32 | 0.00 |
|  |  | timeframe + treatment + floral abundance | 6 | -544.42 | 1101.68 | 222.99 | 0.00 |
|  |  | timeframe * treatment + floral abundance | 1 | -1077.26 | 2156.56 | 1277.87 | 0.00 |
|  |  | intercept | 1 | -1285.62 | 2250.05 | 1363.20 | 0.00 |
|  |  |  |  |  |  |  |  |
|  | Visitation rate | timeframe * treatment * floral abundance | 18 | -879.03 | 396.84 | 0.00 | 0.99 |
|  |  | timeframe + treatment * floral abundance | 14 | -950.48 | 406.43 | 9.95 | 0.01 |
|  |  | timeframe + treatment + floral abundance | 16 | -1442.30 | 407.29 | 10.46 | 0.01 |
|  |  | timeframe * treatment + floral abundance | 26 | -1668.95 | 412.73 | 15.89 | 0.00 |
|  |  | intercept | 13 | -1679.99 | 422.13 | 16.64 | 0.00 |
|  |  |  |  |  |  |  |  |
| High floral abundance | Abundance | timeframe * treatment * floral abundance | 10 | -317.86 | 650.30 | 0.00 | 1 |
|  |  | timeframe + treatment * floral abundance | 8 | -324.76 | 657.72 | 7.42 | 0.00 |
|  |  | timeframe + treatment + floral abundance | 6 | -316.78 | 661.54 | 11.24 | 0.00 |
|  |  | timeframe * treatment + floral abundance | 1 | -323.65 | 661.89 | 11.59 | 0.00 |
|  |  | intercept | 1 | -703.27 | 1412.65 | 762.35 | 0.00 |
|  |  |  |  |  |  |  |  |
|  | Visitation rate | timeframe * treatment * floral abundance | 18 | -1421.77 | 2871.51 | 0.00 | 1 |
|  |  | timeframe + treatment * floral abundance | 14 | -1480.22 | 2975.03 | 103.52 | 0.00 |
|  |  | timeframe + treatment + floral abundance | 16 | -1498.36 | 3011.31 | 139.80 | 0.00 |
|  |  | timeframe * treatment + floral abundance | 26 | -1537.41 | 3083.02 | 211.51 | 0.00 |
|  |  | intercept | 13 | -3497.76 | 7005.84 | 4134.33 | 0.00 |
